# Effective lifestyle interventions to improve type II diabetes self-management for those with schizophrenia or schizoaffective disorder: a systematic review

**DOI:** 10.1186/1471-244X-12-24

**Published:** 2012-03-23

**Authors:** Adriana Cimo, Erene Stergiopoulos, Chiachen Cheng, Sarah Bonato, Carolyn S Dewa

**Affiliations:** 1 Centre for Research on Employment and Workplace Health, Centre for Addition and Mental Health, 455 Spadina, Suite 300, Toronto, Ontario M5S 2G8, Canada; 2 Canadian Mental Health Association, Clinic & Resource Centre, 272 Park Avenue, Thunder Bay, P7B 1C5, Canada; 3 Library Services, Centre for Addiction and Mental Health, 33 Russell Street, Toronto, M5S 2S1, Canada; 4 Department of Psychiatry, University of Toronto, 250 College Street, Toronto, M5T 1R8, Canada

## Abstract

**Background:**

The prevalence of type II diabetes among individuals suffering from schizophrenia or schizoaffective disorders is more than double that of the general population. By 2005, North American professional medical associations of Psychiatry, Diabetes, and Endocrinology responded by recommending continuous metabolic monitoring for this population to control complications from obesity and diabetes. However, these recommendations do not identify the types of effective treatment for people with schizophrenia who have type II diabetes. To fill this gap, this systematic evidence review identifies effective lifestyle interventions that enhance quality care in individuals who are suffering from type II diabetes and schizophrenia or other schizoaffective disorders.

**Methods:**

A systematic search from Medline, CINAHL, PsycINFO, and ISI Web of Science was conducted. Of the 1810 unique papers that were retrieved, four met the inclusion/exclusion criteria and were analyzed.

**Results:**

The results indicate that diabetes education is effective when it incorporates diet and exercise components, while using a design that addresses challenges such as cognition, motivation, and weight gain that may result from antipsychotics.

**Conclusions:**

This paper begins to point to effective interventions that will improve type II diabetes management for people with schizophrenia or other schizoaffective disorders.

## Background

In 2005, the World Health Organization (WHO) predicted that the prevalence of type II diabetes will double by 2030, to affect 366 million people globally [1]. Consequently WHO developed an action plan to increase access to type II diabetes healthcare by 2013 [2]. The increasing rate of this chronic illness is a concern because when glucose (sugar) cannot be absorbed by vital organs, glucose remains in the bloodstream. This leads to persistently high blood glucose levels, which is used as an indicator in diabetes management. Consistently high blood glucose is linked to complications such as cardiovascular disease, blindness, neuropathy, kidney failure, and poor wound healing resulting in infection that may lead to amputation [3].

Not everyone is at equal risk for the development of this chronic illness. Individuals suffering from schizophrenia and schizoaffective disorders are at a greater risk of type II diabetes, with prevalence rates reaching more than two times those of the general population [4-6]. While it has been reported that people with schizophrenia may be genetically predisposed to type II diabetes, several other risk factors could contribute to the development of type II diabetes among people with schizophrenia [7]. Some antipsychotic medications such as olanzapine and clozapine, can cause side effects that promote the onset of type II diabetes [8-10]. These side effects include weight gain, dyslipidemia, hypertension, cardiovascular disease and decreased glucose tolerance [4,6,11]. Furthermore, El-Mallakh [6] observed that poorer health is exacerbated due to high rates of unemployment and reliance on social support, often leaving patients without financial resources to follow dietary guidelines.

Between 2004 and 2005, the debilitating health challenges associated with schizophrenia were recognized by the American Psychiatric Association (APA), American Diabetes Association (ADA), Canadian Diabetes Association (CDA), American Association of Clinical Endocrinologists (AACE), and the North American Association for the Study of Obesity (NAASO) [4,12]. These professional associations recommended continuous metabolic monitoring as a strategy to both prevent and diagnose type II diabetes [4]. Additionally, the need for interdisciplinary care incorporating psychiatrists, psychiatric nurses, family physicians, and diabetes specialists was recognized in response to the complexity of these combined illnesses [12]. However, effective treatment was not included for people with schizophrenia who have type II diabetes.

Given the risk of diabetes-related complications, the ADA and the CDA recommend that all individuals with type II diabetes be provided with Diabetes Self-Management Education (DSME) to successfully control their blood sugar [13,14]. Although DSME that incorporates lifestyle interventions are employed for the general population with type II diabetes, such programs are rarely offered to people who experience schizophrenia and who have type II diabetes. This is reflective of the fact that this population receives poorer diabetes care compared to those without severe mental illness [15,16]. Goldberg et al. [15] found that the frequency of screening and monitoring exams, such as tests for glycated hemoglobin (HbA1c), eye examinations, and identifying serum fat levels, were not meeting the ADA's and CDA's recommendations. Individuals with schizophrenia were also less likely to receive diabetes education. Dickerson et al. [17] found that 48% of participants with severe mental illness had not received diabetes education within the last 6 months. As a result, this population experiences higher rates of hospitalization for hyper or hypoglycaemic episodes, and for infections [18].

Taken together, suboptimal diabetes care quality and the absence of effective treatment recommendations for people who have type II diabetes and schizophrenia or schizoaffective disorders is contributing to a life expectancy that is 20% lower than the general population [18,19]. In response to the need for evidence on this topic, this systematic evidence review (SER) will identify effective lifestyle interventions that enhance quality care in individuals who have type II diabetes and schizophrenia or schizoaffective disorders. Thus, the results from this review can be used to improve type II diabetes management, thereby reducing the burden on the healthcare system from the complications associated with chronically high blood glucose.

## Methods

### Literature search

Electronic searches using Medline, PsycINFO, CINAHL, and ISI Web of Science databases were conducted on June 15, 2011. The search strategies, which are presented in Table 1, were developed in consultation with SB, a librarian scientist. The literature search yielded the identification of 1810 possibly eligible studies. From these, 920 abstracts were retrieved from Medline, 411 from PsycINFO, 88 from CINAHL and 391 from ISI Web of Science.

**Table 1:** Search Terms used for Medline search strategy

Database	Search Terms
Medline	(exp Diabetes Mellitus type 2 OR Diabe*.mp.) **AND **(exp "Quality of Health Care" OR exp. Access to information OR access.mp. OR exp.Evaluation studies as topic OR treatment.mp. OR exp.Early intervention (Education) OR intervention.mp. OR interdiscipl* treatment approach.mp. OR treatment effectiveness evaluation.mp. OR effective intervention.mp. OR evaluat*.mp. OR exp.delivery of health care OR exp.Treatment Outcome OR treatment outcom*.mp. OR clinical practice.mp. OR effective treatment.mp. OR blood glucose self-monitoring/OR exp drug monitoring OR exp.Life Style OR lifestyle intervention.mp. OR exp.Problem Solving OR problem solv*.mp.) **AND **(exp.schizophrenia OR exp.psychotic disorder OR early psych*.mp. OR first episod* psych*.mp. OR first onset psych*.mp, "schizophrenia and disorders with psychotic features" OR early psychosis.mp. OR first episod* psychosis.mp. OR first onset psychosis.mp. OR first onset psych*.mp)

PsycINFO	(Diabete* OR Diabetes OR exp. Diabetes) **AND **(exp Treatment OR intervention OR interdisciplinary treatment approach OR exp Early Intervention OR treatment effectiveness evaluation OR "quality of care" OR effective intervention.mp. OR exp Evaluation OR evaluat*.mp. exp Health Care Delivery OR exp Treatment Outcomes OR treatment outcom*.mp. OR exp Clinical Practice/OR clinical practice.mp.OR effective treatment.mp. OR exp Lifestyle Changes/OR lifestyle intervention.mp. OR exp Problem Solving/OR problem solv*.mp. OR treatment) **AND **(schizo* OR early psych* OR first episod* psych* OR first onset psych* OR exp.schizophrenia OR acute psychosis OR psychosis OR first episode* psychosis.mp.)

ISI Web of Science	(Diabete* OR Diabetes **AND **(effective intervention OR quality care OR access OR evaluation OR health care quality OR quality health care OR treatment outcome OR effective treatment OR clinical practice OR lifestyle intervention OR problem solv* OR intervention OR interdiscplinary treatment approach OR early intervention OR treatment effectiveness evaluation) **AND **(schizo* OR early psychosis OR early psych* OR first episod* psych* OR first episode* psychosis OR first onset psych* OR first onset psychosis)

CINAHL	(Diabet* OR MH Diabetes Mellitus) **AND **(MM.Clinical Effectiveness OR intervention* OR MH.Quality of Health Care OR MM.Quality of Care Research OR quality care OR MH.Access to Information OR access OR health care quality OR MH.Treatment Outcomes OR MM.Outcomes of Education OR MH.Outcomes Health Care OR treatment outcome* OR effective treatment OR MM.Practice Patterns OR clinical practice OR MH.Life Style OR lifestyle intervention OR MH. Problem Solving OR problem solv* OR early intervention OR MM.Program Evaluation OR MM.Glycemic Control or glycemic control OR MM.Self-Efficacy or self*efficacy OR MH.Self Care OR self*care) **AND **(MH.Schizophrenia OR MM.Schizophrenia, Childhood OR MM.schizoaffective Disorder OR MH Psychotic Disorders OR schizo* OR early psych* OR first episod* psych* OR first onset psych*)

### Eligibility assessment

Each title and abstract was independently screened by AC and ES in accordance with the following inclusion criteria that was developed a priori: i) study participants must have a medical diagnosis of both type II diabetes and schizophrenia or schizoaffective disorder, ii) the intervention must target a lifestyle factor associated with diabetes self-care, such as problem-solving skills, education classes, diet or exercise, iii) the outcome measures that determine the success of the intervention must either consider HbA1c, fasting blood glucose (FBG), body mass index (BMI) or weight lost (measured in pounds or kilograms). Interventions that did not exclusively recruit individuals with schizophrenia or schizoaffective disorder were considered. Studies were excluded if they were in a language other than English, French, Italian or Greek; focused on metabolic syndrome, genetics or screening; or considered risk factors for developing type II diabetes without testing an intervention. Abstracts were also read if a title did not provide sufficient information for exclusion. After considering abstracts for eligibility, full text articles were rated for eligibility. In accordance with an inter-rater reliability of 0.40, disagreements between AC and ES were discussed with a third rater, CSD, and a collective consensus was reached. The eligible articles were subsequently rated for quality, and relevant references were considered.

### Quality assessment

A 13-item quality assessment checklist was adapted from Lagerveld et al. [20,21]. Items assessed study design, intervention measurements, outcome measurements, and the presentation of data and analysis. Additional file 1 contains a complete list of items. Each study that met the inclusion/exclusion criteria was independently screened by AC and ES for quality assessment criteria adapted and developed a priori. The inter-rater reliability was 0.62. All disagreements in scoring were discussed and quality rating was reached through consensus between the two independent raters.

Articles that met all quality items were considered excellent. Papers were rated fair and excluded if they had any of the following exclusion criteria: i) does not state the main features of the population which was defined as stating the recruitment location, geographic location, age, gender and eligibility criteria; ii) no account for lifestyle factors during data collection and analysis; iii) follow-up measurements do not include HbA1c, BMI, weight loss, and/or FBG. Therefore, a study was considered good if it did not meet the criteria to be considered fair, but lost other quality points, such as not discussing initial participation rates, and not using appropriate statistical methods or calculating statistical significance.

## Results

### Determining relevant literature

**Figure 1:**
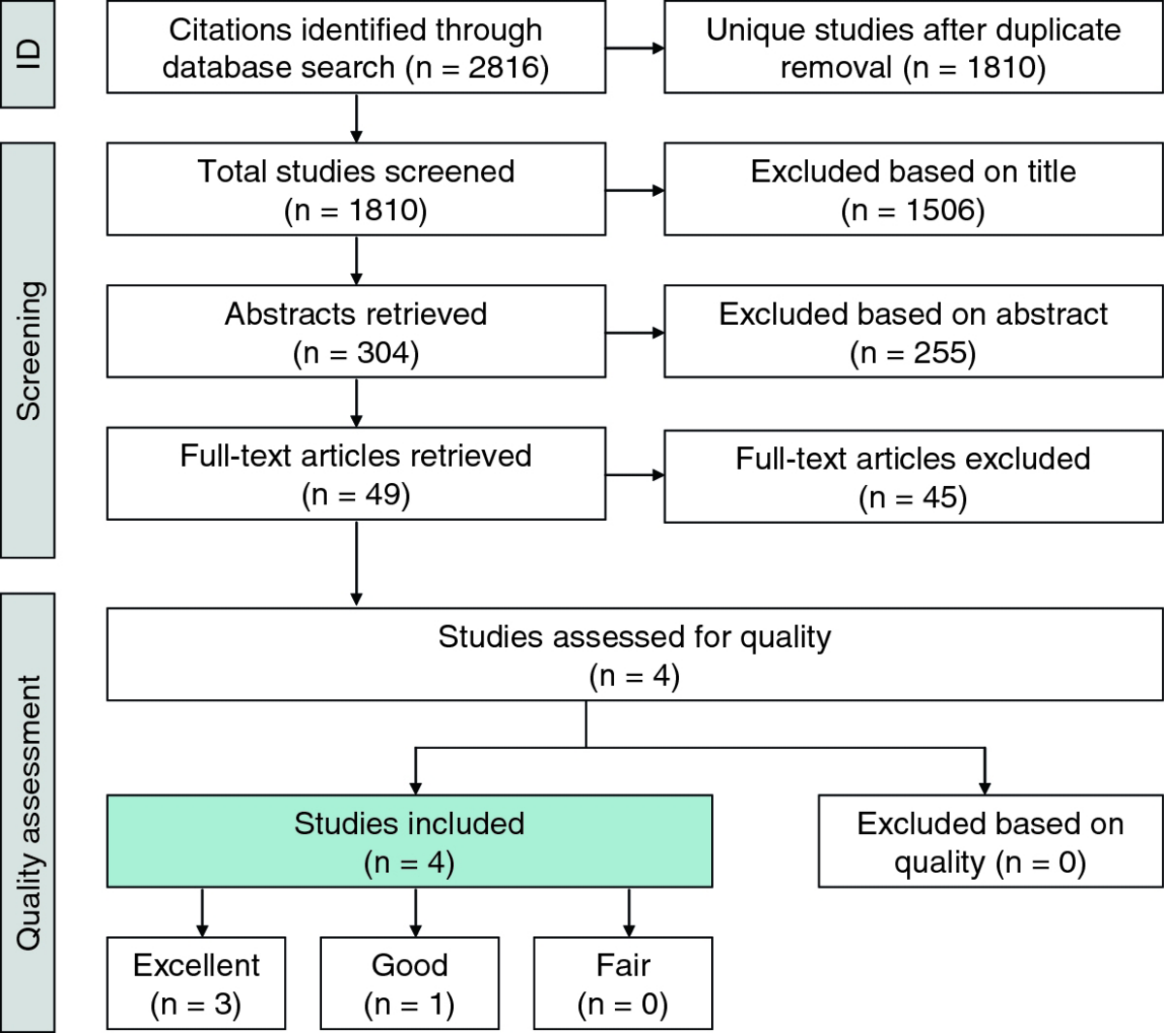
**Flowchart of literature search results and inclusions/exclusions**.

The systematic search of four databases (Medline, PsycINFO, CINAHL, and Web of Science) yielded a total of 1810 unique studies. Raters AC and ES independently screened titles and eliminated those that met the exclusion criteria. A total of 304 abstracts were reviewed in accordance with the inclusion/exclusion criteria. Of these, 49 full-text articles were retrieved. Four articles met the final inclusion criteria. This process of inclusions and exclusions is depicted in Figure 1.

The following is a list of reasons for excluding the 45 full text articles: i) A total of 2.2% (n = 1) was a textbook chapter that elaborated on a study already included; ii) Another 2.2% (n = 1) proposed an intervention that would hypothetically be effective without testing its effectiveness; iii) 4.4% of articles (n = 2) were interventions where outcome measurements did not include blood glucose or weight assessment; iv) A total of 15.6% (n = 7) did not include both type II diabetes and schizophrenia in the study population; v) Another 22.2% of the articles (n = 10) focused on increasing glycaemic control through changing medication, without addressing lifestyle factors; vi) The remaining 53.3% of papers (n = 24) did not assess the effectiveness of an intervention.

### Methodological quality

The four articles that met all inclusion criteria were assessed using the methodological quality assessment checklist that categorized articles as being excellent, good or fair. Of these, three were assessed to be excellent, and the remaining study was rated good. Thus, no articles were excluded at this step, because all met quality criteria good or better.

### Characteristics of included studies

The significant characteristics of each paper are presented in Table 2. Major findings of each intervention analyzed are presented in Table 3. The included articles also shared common elements: each study recruited participants from different US states; they all considered the older-adult population, as indicated by the mean participant age ranging between 44-53 years; interventions each included exercise promotion and nutrition education; they also incorporated components that addressed the challenges associated with this particular population, such as decreased cognitive ability and reduced motivation [22,23]. For example, inpatient modules in Lindenmayer's et al. [24] study were taught for four months before initiating a new level. Some of McKibbin et al.'s [23] strategies involved gradually introducing new topics, utilizing memory aids, and providing minimal text so as to simplify messages. One important difference to note is the variation in study locations: two programs recruited from a psychiatric hospital inpatient setting [24,25]. The remaining two articles addressed health needs of individuals in out-patient mental health settings, such as board-and-care accommodations and community clubhouses [23,26].

**Table 2:** Summary of Article Characteristics

Author	Year	Location	Study Design	Length of Intervention	Sample Size	Mean Participant Characteristics	Location	Type of Intervention
Lindenmayer et al.	2009	US	retrospective, no control group	12 months	275 (72 diabetes)	mean age: 44.25, 78.5% had a psychotic disorder	Inpatient	Psychoeducation

Teachout et al.	2011	US	pre-test post-test, no control group	6 months	13	mean age: 45, 85% had a psychotic disorder,	Inpatient	Diabetes Education

McKibbin et al.	2006	US	RCT	6 months	64	mean age: 53, all had a psychotic disorder	Outpatient	Psychoeducation

McKibbin et al.	2010	US	RCT	12 month follow-up	52	mean age: 52.4, all had a psychotic disorder	Outpatient	Psychoeducation

RCT = Randomized Control Trial

**Table 3:** Intervention Outcome Measurements

Author	Year	Diabetes Knowledge Outcome	Average Weight Loss (lbs)	BMI (Kg/m2) Reduction	Blood Glucose (mg/DL) Reduction	HbA1c (%) Reduction
Lindenmayer et al.	2009	All subjects: Nutrition/healthy lifestyle BL = 17.1/30, change @ 6M = 20.82/30 (*P *< 0.001); Fitness and exercise module BL = 16.97/30, change @ 6M = 20.27/30 (*P *< 0.001)	Diabetes: 5.98 (*P *= nr), Non-diabetes: 4.46 (*P *= nr)	Diabetes: BL = 33.94, change @ 6M = 30.55 (*P *= nr); non-diabetes BL = 29.55, change @ 6M = 27.36 (*P *= nr)	Diabetes: BL = 115.84, change @ 12M = 98.05 (*P *< 0.001); non-diabetes: BL = 92.81 change @ 12M = 87.69 (*P *= nr)	

Teachout et al.	2011		20.35 (P = nr)		40% in the recommended range of 90-110	

McKibbin et al.	2006	DART: BL = 0.5, change @ 6M = 0.7 (*P *< 0.001); UC: no change (*P *< 0.001)	DART: 5.1 (*P *< 0.001); UC: -6.8 (*P *< 0.001)	DART: BL = 33.6, change @ 6M = 32.9 (*P *< 0.001); UC: BL = 32.9, change @ 6M = 33.9 (*P *< 0.001)	DART: BL = 163.9, change @ 6M = 125.7 (P = ns) UC: BL = 147.2, change @ 6M = 143.4 (*P *= ns)	DART: BL = 7.4, change @ 6M = 6.9; (*P *= ns) UC: BL = 6.7, change @ 6M = 6.8 (*P *= ns)

McKibbin et al.	2010	DART: BL = 0.5, change @ 6M = 0.6 (*P *< 0.01); UC: no change (*P *< 0.01)		DART: BL = 33.9, change @ 6M = 32.9 (*P *< 0.01); UC: BL = 32.6, change @ 6M = 34.0 (*P *< 0.01)		DART: BL = 7.3 change @ 12M = 6.9 (*P *= ns); UC: BL = 6.8, change @ 12M = 7.9 (*P *= ns)

BL = Baseline measurement; 6M = 6 month follow-up; 12M = 12 month follow-up; DART = Diabetes Awareness and Rehabilitation Training experimental group; UC = Usual Care control group; nr = not reported; ns = not statistically significant

### Results of in-patient interventions

Both in-patient programs provided information regarding the importance of exercise, and provided strategies for making this lifestyle change [24,25]. After enhancing patient motivation through classes, achieving physical activity recommendations in both studies were promoted by providing exercise facilities. Lindenmayer et al.'s [24] knowledge assessment of the four month fitness and exercise module demonstrated an improvement in scores, as they increased from 56.6% to 67.6% (*P *< 0.001). Participants in Teachout et al.'s [25] intervention were given additional resources: pedometers, encouragement to walk, and yoga classes. Fitness knowledge was not assessed following the intervention.

In both studies, nutrition lessons were aimed at improving dietary habits. In Lindenmayer et al.'s [24] program, workshops provided dietary tips and a weekly 25 dollars was given to enable the purchase of healthy foods. This strategy was successful as the knowledge assessment scale for the four month section on nutrition and healthy lifestyle increased from 57% to 69.4% (*P *< 0.001). Teachout et al.'s [25] method involved modules on healthy meal planning, shopping and food preparation over a period of six months. However, participant knowledge gained from this approach was not measured.

Overall, the psychiatric in-patient interventions had a positive impact on weight, BMI and blood glucose measurements, thus indicating the effectiveness of combining diet and exercise. After 12 months, participants with type II diabetes and severe mental illness in Lindenmayer et al.'s [24] trial lost a mean total of 5.98 lb. BMI was also reduced from 33.94 kg/m2 to 30.55 kg/m2 (statistical significance was not calculated for BMI or weight loss). Furthermore, blood glucose also decreased significantly, from an average of 115.85 mg/DL to 98.05 mg/DL (*P *< 0.001). This reduction was significantly related to the nutrition module that was completed during the beginning of the intervention. Similarly, all of Teachout et al.'s [25] participants reduced their weight, with an average loss of 20.35 lb, and 40% of fasting glucose levels met the recommended value as outlined by the ADA (statistical significance was not calculated).

### Results of out-patient interventions

In McKibbin et al.'s [23] six month Diabetes Awareness and Rehabilitation Training (DART), participants received 90-min weekly sessions providing diet, exercise and other diabetes self-care strategies such as monitoring blood glucose levels. Physical activity modules involved learning about the different types of exercise, how being active can impact blood glucose, and how to keep track of daily exercise. Participants were also given pedometers, encouraged to walk, and tracked weekly weight changes to increase motivation.

Participants additionally received simplified nutrition education that provided knowledge of the different food groups, adequate portion sizes, healthy meal planning and label reading, as well as substituting sugar consumption with fat and fibre.

Comparing the outcome measurements of the DART program with a Usual Care (UC) group that received pamphlets from the ADA and continued seeing their family physician indicated the effectiveness of the DART intervention. While the control group gained a total of 6.8 lbs during the study period, intervention participants lost an average of 5.1 lbs (*P *< 0.001). Correspondingly, DART participants' BMI was reduced on average from 33.6 kg/m2 to 32.9 kg/m2, whereas the UC increased from 32.9 kg/m2 to 33.9 kg/m2 (*P *< 0.001). Study participants also enhanced their diabetes knowledge from 0.5 to 0.7, while there was no change in the scores for the control group (*P *< 0.001). Although there was a reduction in fasting blood glucose and HbA1c in both the experimental and control group, findings were not statistically significant.

The effectiveness of this intervention was measured six months after the end of the study [26]. The DART group maintained their average BMI of 32.9 kg/m2, while the control group continued to gain weight, totaling an average of 34 kg/m2. Although diabetes knowledge dropped at 12 months from 0.7 to 0.6, it was still higher than the baseline value of 0.5. Therefore, the follow-up study revealed that sustainable skills were gained from the DART intervention.

## Discussion

The current literature assessing the management of type II diabetes for individuals with schizophrenia and schizoaffective disorders has indicated that there are a number of tactics used to manage blood glucose levels. One that has drawn attention involves changing the antipsychotics prescribed. Antipsychotics have been the focus because of the weight gain and glucose intolerance that is associated with use of these medications [4,5,11]. A meta-analysis conducted by Barnett et al. [10] reported that patients treated with clozapine and olanzapine have higher rates of weight gain and therefore increased diagnosis of type II diabetes compared to other antipsychotics. Consistent with these findings are case studies that changed antipsychotic medications as an intervention to manage blood glucose in type II diabetes. Lerner et al. [27] lowered the dose of olanzapine for two patients and noted a reduction in blood glucose levels. Furthermore, a total of four case studies observed a remission of type II diabetes upon the replacement of olanzapine with risperidone, as HbA1c levels normalized [28-31]. Given the impact of antipsychotic medications in the development of this disease, recommendations are often made to change prescriptions if persistent weight gain and onset of type II diabetes occurs [10,12,32]. However, this treatment approach poses challenges because individuals with schizophrenia may experience a relapse of psychotic or depressive symptoms during the transition period between medications [33,34]. Moreover, not every individual has a therapeutic response to all antipsychotics [35].

Considering the challenges with changing antipsychotic medication, our SER aimed at determining effective delivery of diet and lifestyle interventions to enable management of type II diabetes in individuals with schizophrenia or schizoaffective disorders. The success of diet and lifestyle interventions to prevent or manage type II diabetes in individuals with and without schizophrenia or schizoaffective disorders has been documented in the literature. Menza et al. [36] conducted a 12-month lifestyle intervention that combined diet and physical activity in patients with schizophrenia and schizoaffective disorders. Findings included reduced weight and BMI, increased nutritional knowledge, and improved HbA1c levels, thus minimizing risk of type II diabetes development. Torgerson et al. [37] was successful in preventing the onset of type II diabetes in obese individuals by combining weight loss with the inhibition of an enzyme that breaks down fats. Additionally, Lim et al.'s [38] paper suggests that weight loss enabled by restricting calories to 600 kcal/day is an effective method to decrease BMI within the general type II diabetes population (*P *< 0.05). Calorie restriction also corresponded to greater blood glucose control, as glycated hemoglobin decreased to normal levels in 8 weeks (*P *< 0.05).

While the relationship between lifestyle interventions with weight loss and improved glycated hemoglobin is well documented, the most effective way of delivering DSME is not always clear for individuals with schizophrenia or schizoaffective disorders. Therefore, our paper makes an important contribution to the literature by highlighting effective DSME strategies to support the integration of healthy habits into lifestyle. Each DSME lifestyle intervention reviewed in our paper observed reduced weight and BMI in the presence of intervention strategies that addressed the challenges associated with schizophrenia, such as decreased cognitive ability, reduced motivation and limited access to resources [6,22,23]. Additionally, Lindenmayer et al. [24] and Teachout et al. [25] observed that diet can reduce fasting blood glucose.

One drawback consistent in all interventions was the absence of finding statistical significance when HbA1c levels were reduced. One likely explanation is the fact that interventions were not long enough to observe changes in HbA1c levels [23,39]. This is because retrieving an accurate measure may require blood glucose to be controlled for more than three months. If lifestyle changes were not fully adopted, it would be more difficult to see a reduction in HbA1c, even if some changes occurred. In contrast, blood glucose measures depict blood glucose at one point in time and this value can fluctuate hourly. Thus, HbA1c is considered a more reliable measure of overall blood glucose control. Additionally, HbA1c has been correlated with diabetes complications, while blood glucose has not [39].

Because all quality ratings of the analyzed studies resulted in excellent and good assessments, there is a strong level of evidence to support our conclusion made in this SER [40]. However, while the findings of this paper indicate that lifestyle interventions positively impact type II diabetes management, there were limitations associated with the heterogeneity of the study settings included in the analysis. In the psychiatric inpatient setting, participation in the interventions was structured in the individual's daily routine. Conversely, in McKibbin et al.'s [23] study, recruitment from community clubhouses and board-and-care facilities indicates that participant involvement required a greater level of self-motivation. Therefore, care needs to be taken if inpatient interventions are adapted in outpatient settings, because individuals within the community may have limited access to fitness resources, such as gym equipment and safe areas to exercise.

As a result of limiting searches to four databases containing primarily peer-reviewed material, a potential publication bias is an additional limitation of this SER. However, the databases searched covered an extensive scope of clinical disciplines that were relevant for the nature of the research question: PsycINFO captures psychological literature, CINAHL retrieves the nursing and allied health, Medline contains medical literature, while ISI Web of Science is multidisciplinary. Additionally, while grey literature was not searched for directly, PsycINFO includes doctoral theses available in Dissertations Abstracts International. Additionally, due to a lack of fluency in languages other than English, French, Italian and Greek, papers in other languages were not considered.

While the analyzed studies indicate the short-term effectiveness of lifestyle interventions in individuals with type II diabetes and schizophrenia, the long-term sustainability of treatment outcomes has not been explored. Additionally, it has been observed that such lifestyle interventions are effective for older adults with type II diabetes and schizophrenia. However, the success of such approaches within the first episode population is unknown. Although adults over 40 years of age are an important population to consider due to the increased risk factors of diabetes from long-term use of antipsychotics and being over the age of 45, the onset of type II diabetes in individuals aged 20 to 49 years is increasing [12,41,42]. Therefore, an additional recommendation for future research involves determining the effectiveness of such programs for youth and young adults experiencing early-onset psychosis who also have type II diabetes.

Overall, the current state of the literature suggests promise within this line of inquiry. However, our review also indicates that there are gaps in the current literature. Of additional significance is the need for interdisciplinary teams when addressing the complex health concerns associated with both schizophrenia and type II diabetes [12].

## Conclusion

Findings of this SER suggest that lifestyle interventions can be effective in managing type II diabetes in patients that concurrently have schizophrenia or schizoaffective disorders. However, they should be sensitive to the unique challenges associated with type II diabetes and schizophrenia. These challenges include decreased cognition and motivation, limited resources, as well as negative side effects (i.e. loss of energy and weight gain) that may result from antipsychotics. Successful strategies involved positive encouragement to make changes, gradual introduction of new concepts and skills relating to diet and exercise, conveying simple messages for complex topics such as food skills relating to cooking and planning meals, and incorporating memory aids [23,24].

The findings of this review indicate a strong level of evidence; there are effective interventions for people with schizophrenia and type II diabetes. However, there is a need for continued research to fill gaps. These include exploration of the long-term sustainability of DSME lifestyle interventions, and addressing the young adult population suffering from schizophrenia or schizoaffective disorders with type II diabetes. Furthermore, the need for interdisciplinary interventions should be kept in mind, as the collective expertise of psychiatrists, psychiatric nurses, family physicians and diabetes educators such as dietitians are all needed. Taking these factors into account will enable policies and interventions to be developed that improve the healthcare and quality of life of individuals with type II diabetes and schizophrenia or schizoaffective disorders.

## Competing interests

The authors declare that they have no competing interests.

## Authors' contributions

AC led the conception, design, data acquisition, analysis and interpretation of the data. ES collaborated on the design, data acquisition and analysis. CC collaborated on the design and acquisition of data. SB collaborated on the design and data acquisition. CSD collaborated on the conception, design and acquisition of data, and supervised the data analysis and interpretation. All authors read and approved the final manuscript.

## Pre-publication history

The pre-publication history for this paper can be accessed here:

http://www.biomedcentral.com/1471-244X/12/24/prepub

## Supplementary Material

Additional file 1 **Quality assessment checklist; The additional file contains the quality checklist criterion that was used to determine the quality of the papers being analyzed for the systematic review**. Scores of each article are displayed as well as the quality checklist items that were adapted from Lagerveld et al. [20]. Click here for file
