# Supplementary material for: Effective lifestyle interventions to improve type II diabetes self-management for those with schizophrenia or schizoaffective disorder: a systematic review
Source: BMC Psychiatry. 2012 Mar 23;12:24. doi: 10.1186/1471-244X-12-24 (PMC3340308; doi:10.1186/1471-244X-12-24)
Supplement: Additional file 1 — Quality assessment checklist; The additional file contains the quality checklist criterion that was used to determine the quality of the papers being analyzed for the systematic review. Scores of each article are displayed as well as the quality checklist items that were adapted from Lagerveld et al. [20]. [file 1471-244X-12-24-S1.DOC]

**Quality Assessment Checklist**

| **Paper** | **1** | **2** | **3** | **4** | **5** | **6** | **7** | **8** | **9** | **10** | **11** | **12** | **13** | **TOTAL** |
| --- | --- | --- | --- | --- | --- | --- | --- | --- | --- | --- | --- | --- | --- | --- |
| McKibbin et al. 2009 | 1 | 1 | 1 | 1 | 1 | 1 | 1 | 1 | 1 | 1 | 1 | 1 | 1 | 13 |
| Teachout, et al. 2011 | 1 | 1 | 1 | 0 | 1 | 1 | 1 | 1 | 0 | 0 | 1 | 1 | 0 | 9 |
| McKibbin et al. 2010 | 1 | 1 | 1 | 1 | 1 | 1 | 1 | 1 | 1 | 1 | 1 | 1 | 1 | 13 |
| Lindenmayer et al., 2009 | 1 | 1 | 1 | 1 | 1 | 1 | 1 | 1 | 1 | 1 | 1 | 1 | 1 | 13 |

Quality assessment criterion:

1 = Positive if the study has a control group that is similar to the experimental group

Such as age, Axis I diagnosis, education level, A1C reading, and BMI measurement
 pre-intervention

2 = Positive if the intervention was described well enough for the reader to have an
 understanding of what the intervention entailed
 Ex. Describing the elements of an education program plan, the channel through which
 instructions were given to participants (ie. Web, telephone, in person), types of educational
 material that were given to participants

3 = Positive if the main features of the study population are stated
 Such as, recruitment location, geographic location, age, gender, eligibility criteria

4 = Positive if there is mention regarding initial participation rates

5 = Positive if methods of data collection were stated

6 = Positive if the researchers accounted for lifestyle factors during data collection and analysis
 Lifestyle factors include diet modifications, exercise, diabetes education knowledge that
 are necessary for self-care activities like foot examinations and checking blood glucose

7 = Positive if follow-up measurements, such as HbA1C, FBG or BMI, are stated
 Give point as long as at least one of the above mentioned criteria are stated

8 = Positive if the rates of attrition are identified at each outcome measurement during the
 longitudinal study

9 = Positive if any confounders are mentioned and accounted for in the analysis

10 = Positive if the statistical model used is appropriate for the outcome studied
 - t-test used for continuous variables
 - chi-square used for non-continuous variables
 - ANOVA used when there are more than three continuous variables

11 = Positive if authors answered their research question

This will be determined by identifying the research goals at the beginning of the paper,
 and ensuring that the results and discussion are in accordance with them

12 = Positive if there is any discussion of study limitations (including biases), and how the
 study may have been affected as a result

13 = Statistical significance of association, as indicated by assigning a P-value of less than 0.5, are tested and relevant parameters are presented
